# Climate Change Habitat Model Forecasts for Eight Owl Species in the Southwestern US

**DOI:** 10.3390/ani13243770

**Published:** 2023-12-06

**Authors:** Jean-Luc E. Cartron, F. Jack Triepke, Dale W. Stahlecker, David P. Arsenault, Joseph L. Ganey, Charles D. Hathcock, Hunter K. Thompson, Matthieu C. Cartron, Kenneth C. Calhoun

**Affiliations:** 1 Department of Biology, University of New Mexico, Albuquerque, NM 87131, USA; 2 U.S. Department of Agriculture, Forest Service, Southwestern Region, Albuquerque, NM 87102, USA; 3 Eagle Environmental, Inc., Santa Fe, NM 87508, USA; 4 American Valley Environmental, LLC, Quincy, CA 95971, USA; 5 U.S. Department of Agriculture, Forest Service, Rocky Mountain Research Station, Flagstaff, AZ 86001, USA; 6 Chiricahua Desert Museum, Rodeo, NM 88056, USA; 7 Colorado School of Mines, Golden, CO 80401, USA; 8 Department of Statistics, North Carolina State University, Raleigh, NC 27695, USA; 9 Daniel B. Stephens & Associates, Albuquerque, NM 87109, USA

**Keywords:** *Aegolius acadicus*, *Aegolius funereus*, *Asio otus*, *Bubo virginianus*, climate change, ecosystem vulnerability model, *Glaucidium gnoma*, habitat model forecasts, *Megascops kennicotti*, *Megascops trichopsis*, *Psiloscops flammeolus*

## Abstract

**Simple Summary:**

We used pre-1990 climate envelopes and an ecosystem vulnerability model to forecast shifts in the extent of breeding habitats for eight owl species occurring in montane woodlands and forests of the southwestern US. For five of the eight species, the regional habitat extent was projected to decline by at least 60% by 2090. The steepest rates of habitat loss were predicted for the boreal owl (*Aegolius funereus*) and the flammulated owl (*Psiloscops flammeolus*). The boreal owl, which is absent in Arizona and at the trailing edge of its distribution in New Mexico, was projected to retain only about 15% of its mapped regional habitat by 2090. Generalist and lower-elevation owl species fared better in our model predictions, while the whiskered screech-owl (*Megascops trichopsis*) habitat was projected to contract locally yet expand to the north. The results of this study suggest high exposure to climate change impacts for Southwestern North America’s upper-elevation owls. Long-distance migration and low philopatry may prove important to some montane owl populations in adapting to the regional loss of montane woodland and forest habitats.

**Abstract:**

The high-resolution forecasting of vegetation type shifts may prove essential in anticipating and mitigating the impacts of future climate change on bird populations. Here, we used the US Forest Service Ecological Response Unit (ERU) classification to develop and assess vegetation-based breeding habitat profiles for eight owl species occurring in the foothills and mountains of the Southwestern US. Shifts in mapped habitat were forecast using an ecosystem vulnerability model based on the pre-1990 climate envelopes of ERUs and the Intergovernmental Panel on Climate Change’s (IPCC) A1B moderate-emission scenario for the future climate. For five of the eight owl species, the regional breeding habitat extent was projected to decline by at least 60% by 2090. Three species, the boreal owl (*Aegolius funereus*; at the trailing edge of its distribution), flammulated owl (*Psiloscops flammeolus*), and northern pygmy-owl (*Glaucidium gnoma*), were projected to experience the steepest habitat loss rates of 85%, 85%, and 76%, respectively. Projected vegetation shifts overlaid with well-documented flammulated owl breeding populations showed the complete or near complete loss of habitat by 2090 in areas of montane forest currently supporting dense aggregations of owl territories. Generalist or lower-elevation owl species were predicted to be less impacted, while, for the whiskered screech-owl (*Megascops trichopsis*), the contraction of the current habitat was nearly offset by a projected northward expansion. In general, the results of this study suggest high exposure to climate change impacts for the upper-elevation forest owls of semi-arid Southwestern North America. Long-distance migration and low natal philopatry may prove important to some montane owl populations in adapting to the regional loss of habitat.

## 1. Introduction

If left unchecked, anthropogenic climate change could soon result in the extinction of one in six species on Earth [1]. To minimize global (and regional) biodiversity losses, there is an urgent need to identify both the species at risk and the extent of their vulnerability [2,3]. A useful framework in assessing vulnerability to climate change relies on the three concepts of exposure, sensitivity, and adaptive capacity [3]. Exposure is defined as the degree to which a species may experience a change in its surrounding environment, whether from temperature or precipitation shifts, altered vegetation cover, or the disruption of trophic interactions. Sensitivity relates to the species’ dependence on specific environmental or ecological conditions that are projected to change [4,5]. Adaptive capacity is the ability of a species to persist under changing conditions through dispersal, migration, or other adaptation mechanisms [6,7].

Several approaches have been proposed to assess climate change risks and vulnerabilities in bird populations. Already measurable and/or predicted climate change impacts on owls in particular have been studied using demographic models based on simulated weather variables and their effect on population vital rates, either directly or through their interplay with trophic interactions, e.g., [8,9]. In high Arctic Greenland, snowy owl (*Bubo scandiacus*) reproductive success and population densities were predicted to decline due to ongoing demographic shifts (i.e., collapsing of the high-amplitude population cycle) in the main prey, the collared lemming (*Dicrostonyx groenlandicus*) [10,11]. Similarly, stochastic demographic modeling showed that already observable dampening vole prey cycles may be driving a tawny owl (*Strix aluco*) population from Northern England towards extinction [8]. At much lower latitudes, in Switzerland, Jenouvrier [12] projected a dramatic barn owl (*Tyto alba*) population decline under a scenario of non-linear relationships between climate change and population vital rates. During past extreme harsh winters, recorded only twice in a 58-year monitoring period, adult and juvenile barn owl survivorship plummeted due to the extended snow cover duration, reducing small mammal prey availability [13]; under the scenario of non-linear climate change, the incidence of extreme harsh winters would increase, with a direct impact on long-term barn owl stochastic growth rates [12]. In the Southwestern US, negative associations between warm, dry conditions and spotted owl (*Strix occidentalis*) fecundity led to predictions of rapid population declines and a much higher probability of extinction under three emission scenarios compared to current climate conditions [9]. Compared to other birds, spotted owls have relatively narrow neutral thermal zones as they are seemingly less heat-tolerant [14,15].

Here, we provide a new approach to predicting impacts on owl populations at a local and regional scale based on the Intergovernmental Panel on Climate Change’s (IPCC) A1B moderate IPCC emission scenario of the future climate [16] and an ecosystem-based vulnerability assessment developed for the Southwestern US (i.e., the two states of Arizona and New Mexico) [17]. The vulnerability assessment is represented by the level of departure for the late 21st century climate from the characteristic pre-1990 climate envelope of the ecosystem type at each given location, resulting in a probability surface of climate impacts for the two-state area [17]. This emission scenario is considered a plausible balance of expected technological and energy development and a standard representative for mid-range climate forcing and climate research [16,17].

**Figure 1:**
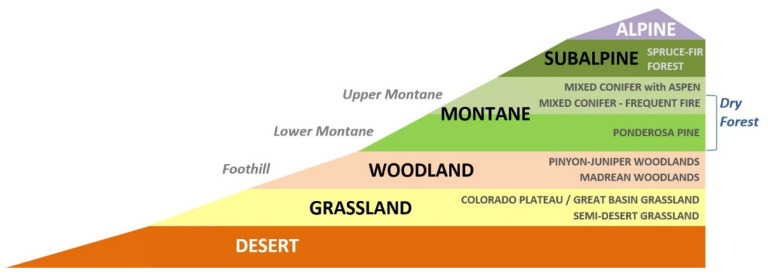
Elevational zonation of vegetation types in the Southwestern US showing desert and grassland at lower elevations and woodlands and forests at mid- to upper elevations.

Land cover in Southwestern North America has changed dramatically since the last ice age, when climate conditions were significantly cooler and more humid and the vegetation consisted largely of coniferous forests and woodlands, all as recently as 12,000 years ago [18,19]. Xerophytic vegetation types would have been limited in extent and concentrated in the southernmost areas of the region. The abundance and locations of ecosystem types found today in Southwestern North America reflect a warmer and drier climate than that 12,000 years ago, the result of natural change over time [20]. High topographic relief has facilitated the conspicuous elevational zonation of the vegetation, with xerophytic vegetation (e.g., grassland, shrubland, and desert scrub) at lower elevations grading into more mesic plant communities (woodlands and forests) at upper elevations (Figure 1). A number of owl species breed in Southwestern North America in association with these woodlands and forests, which, under increasingly warmer and drier conditions, are likely to retreat farther up the elevational gradient. Here, we hypothesize that the montane woodland and forest owls of the Southwestern US are facing high exposure to ongoing and projected climate change. A testable prediction of our hypothesis is that, for at least some of these owl species, the extent of their regional breeding habitat will contract by 2090 under the (IPCC) A1B moderate-emission scenario. The model based on the climate envelopes of individual ecosystem types and the magnitude of departure from these envelopes allows quantitative projections of vegetation-based habitats by the late 21st century [21]. This type of analysis has implications for arid Southwestern North America and similar environments elsewhere in the world.

## 2. Materials and Methods

### 2.1. Habitat Profiles and Spatial Rendering

Spatial models were developed to represent the approximate extent and distribution of breeding habitats for eight species of owls in the montane woodlands and forests of the Southwestern US: the flammulated owl, western screech-owl, whiskered screech-owl, great horned owl, northern pygmy-owl, long-eared owl, boreal owl, and northern saw-whet owl.

As a first step, breeding habitat profiles were created for each of these species based on a literature review and expert opinion and using the 2017 version of the Ecological Response Units (ERU) [22], with each ERU being similar in vegetation potential and plant dominants, historical fire regime, plant succession, and key ecological processes (Table 1). Vegetation provides the basic structure and the primary functions for ecosystems and species habitats [23]. Vegetation is a major environmental factor influencing habitat selection in bird species [24,25], while the high-resolution mapping of vegetation cover is also essential to model the distribution of populations and improve the management of breeding habitats [26]. Not included in our analysis was the Mexican spotted owl (*Strix occidentalis lucida*), a threatened subspecies of the spotted owl occurring in the Southwestern US. Although the Mexican spotted owl is a montane owl, extensive research on this subspecies shows that it selects its habitat based on topography and/or canopy closure rather than vegetation type [27,28].

Of the eight owl species, five have their distribution in the Southwestern US restricted to wooded foothills and mountains during the breeding season, e.g., [29,30], and the habitat profiles used for this study approximated the totality of their regional breeding habitat distribution in 2017. To varying extents, western screech-owls, great horned owls, and long-eared owls also breed in floodplain riparian woodlands and in other low-elevation vegetation types not found in foothills and mountains [29,30]. The montane habitat profiles of these three species therefore represented only partial representations of their total habitat distributions in the Southwestern US. We excluded pinyon–juniper woodland and Madrean pinyon–oak woodland from the habitat profiles of the flammulated owl and the northern saw-whet owl as the relatively small number of breeding records from these vegetation types tended to be restricted to the ecotone with ponderosa pine forest, with denser and taller pinyon pines (*Pinus* spp.) and junipers (*Juniperus* spp.) and scattered ponderosa pines (*Pinus ponderosa*) [31,32].

**Figure 2:**
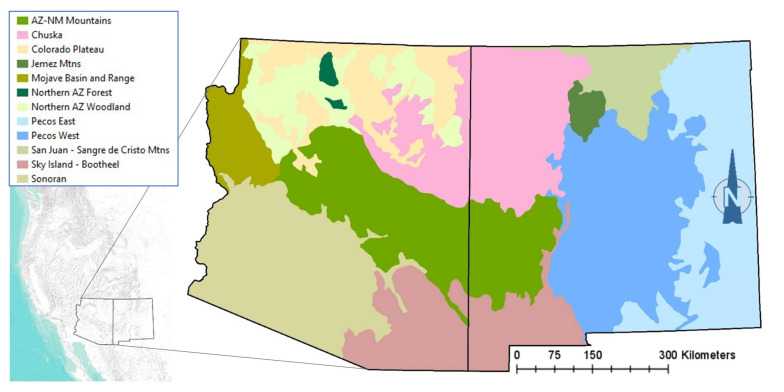
Model zones used for owl habitat profiles and alignment with reported range distributions.

The spatial rendering of each profile (our spatial model) was performed in GIS using state boundaries to focus on Arizona and New Mexico, a geodataset of all Ecological Response Units and their climate vulnerability forecasts [17], and a layer of Ecological Subregions [33] to constrain the species biogeography. Figure 2 shows the model zones used to geographically limit the habitat profiles for some species, with each zone composed of one or more Subregions.

### 2.2. Ecosystem Vulnerability Model and Projections of Future Habitat Distribution

Projections of future habitat distribution were based on measured shifts in climate conditions, from past to future, and expected changes in vegetation pattern based on suitable climate conditions among ERUs and their known climate envelopes [17]. Climate envelopes were built with five climate variables (e.g., Julian date where the sum of degree-days >5 °C reaches 100 degree-days >5 °C based on mean monthly temperature) found to optimize the class separation of ERUs and selected from over 20 variables, including several indices corresponding to combinations of temperature and precipitation measurements [17]. The following equation was used to assign the vulnerability of an ERU at a given location.

(1) $$\mathrm{VS}=\left.\left(\;\frac{\left|x¯\_{Val}_{seg}\right|}{2s}\right.\right)$$

where VS is the vulnerability score for a polygon, x¯ is the mean of one pre-1990 climate variable, Val_seg_ is the year 2090 value for the variable and polygon segment, and *s* is the inter-annual, pre-1990 climate standard deviation for the ERU [17].

The same climate variables used to develop the underlying vulnerability surface were applied to determine the most likely outcomes in the future distribution of ERUs, according to forecast climate exposure relative to the conditions represented by each envelope. Multiple studies from the region and the Western US corroborate the directional change in vegetation patterns anticipated with warmer and drier conditions, including elevational shifts [34,35,36,37] and associated changes in plant composition [17,38,39]. For instance, tree species recruitment in ponderosa pine communities with the greatest climate departure projections are showing an increased probability of the regeneration of pinyon and juniper species, components of warmer–dryer downslope affinities [17]. Vegetation type projections sometimes indicate more than one possible outcome due to overlap among some climate envelopes. In these cases, the interpretation of future distribution erred towards inclusion for any given habitat profile. For upland shrub types (not selected in any breeding habitat profile, but present in the woodland and montane zones), a specific rule was applied to narrow the potential transitions of shrub types to other shrub vegetation types, given the importance of site factors (especially slope, aspect, edaphic properties), shrub recruitment strategies, and natural fire regimes in perpetuating shrub dominance [40,41]. Thus, no shrub types were projected to convert to woodlands and forests under climate model projections. However, woodlands, at their lower ecotone, could be projected to grassland or shrubland types (e.g., Colorado Plateau/Great Basin Grassland, Intermountain Salt Scrub).

Our analyses were restricted to mid-to-upper elevation ecosystem types and did not include lowlands, including desert areas, for which some uncertainty remains regarding climate envelopes. Samples for the four desert ERUs (Chihuahuan Desert Scrub, Chihuahuan Salt Desert Scrub, Mojave–Sonoran Desert Scrub, and Sonora–Mojave Mixed Salt Desert Scrub) were available only from the northern extents of the Chihuahuan and Sonoran provinces, in turn possibly skewing the climate envelopes and overpredicting the vulnerability [17]. All four of these systems are especially resistant to stress and drought extremes, as well as to variability in environmental conditions across temporal scales [42,43,44].

For all species, current and future habitat models were parameterized conservatively, erring towards commission, by the inclusion of vegetation types and model zones. In the case of the whiskered screech-owl, a Central American species whose range extends just into the southern borderlands of Arizona and Mexico, an allowance was made for the future projection and potential northward expansion of the species by adding two model zones.

### 2.3. Habitat Model Validation Datasets

Habitat model validation was performed using a total of 834 locational data points corresponding to georeferenced museum specimen records, i-Naturalist research-grade verified observations (non-duplicate locations only), and owl or owl territory detections in survey or monitoring datasets. All records with precision > 1 km were excluded. Other than western and whiskered screech-owls, known to remain on their territories year-round, museum specimen records were restricted to the months of May–July to avoid the inclusion of data points during migration. i-Naturalist records used for habitat model validation consisted of those during the same period of May–July and/or based on breeding pair, nest, or fledgling photos or recorded singing. Owl surveys were conducted during the May–July period and detections typically involved hearing territorial calls. Our occurrence data do not represent a statistical sample; however, these records provide a sense of the data quality of habitat mapping and the degree of model omission and commission. A habitat buffer of 1 km was used to account for uncertainty in both the habitat mapping and occurrence records. Owl survey datasets were also used to investigate projected shifts in vegetation conditions in areas with documented owl populations or the inference of higher-than-average owl densities. Though typically limited in terms of their spatiotemporal coverage, survey datasets consist of presence–absence data, found to perform better in predictive models than opportunistic records and other presence-only data [45]. At a local scale at least, the owl survey datasets likely improved the accuracy of our assessment of the projected climate-induced impacts on the extent of habitats for some owl species.

#### 2.3.1. Boreal Owl Surveys (New Mexico)

Boreal owls were recorded during standardized, conspecific playback surveys in Northern New Mexico during 1989–1993 [46]. Documented or suspected breeding locales were all revisited in 2012, with additional sites also surveyed [47]. All detections occurred in just three mountain ranges [46,47]. The high rate of detection success at revisited sites in 2012 suggested long-persisting populations [47]. For this study, all documented boreal owl territories were overlaid on maps showing the current and predicted late 21st century distribution of vegetation types generalized from ERUs. The percentage of territories included within our habitat model under current and projected conditions was calculated in ArcGIS.

#### 2.3.2. Flammulated Owl Surveys (New Mexico)

From 1996 through 1998, systematic flammulated owl (*Psiloscops flammeolus*) surveys were conducted between 2000 and 2700 m in the Jemez, Zuni, Magdalena, and San Mateo mountains, and in the Black Range, all in Western New Mexico [32]. Additional surveys were completed through 2006 and in 2011 for the Oso Ridge flammulated owl population in the Zuni Mountains (D. Arsenault, unpubl. data). All surveys involved the search for flammulated owl territories during the nesting season, and nest and territorial male locations were determined with accuracy of ≤3 and 10 m, respectively. The surrounding vegetation consisted primarily of ponderosa pine and Gambel oak (*Quercus gambelii*) forests with herbaceous understories and scattered shrubs; remnant groves of narrowleaf cottonwood (*Populus angustifolia*) in some riparian areas; and ponderosa pine mixed with quaking aspen (*Populus tremuloides*) and Douglas fir (*Pseudotsuga menziesii*) at upper elevations and with pinyon pine (*Pinus edulis*) at mid-elevations [32]. From 1996 through 1998, a total of 86 occupied territories were documented and georeferenced along 149 km of transects [32]. The total number of occupied nesting territories reached 148 with the later surveys in the Zuni Mountains. Owls were detected in montane forest at elevations ranging from 2260 m (2070 m in the Magdalena Mountains) to 2680 m. All documented flammulated owl territories were overlaid on maps showing the current and predicted late 21st century distribution of vegetation types generalized from ERUs. The percentage of territories included within our habitat model under current and projected conditions was again calculated in ArcGIS.

#### 2.3.3. Quadrat Owl Detections (Arizona and New Mexico)

Location data on flammulated owls, western screech-owls (*Megascops kennicottii*), great horned owls (*Bubo virginianus*), northern pygmy-owls (*Glaucidium gnoma*), long-eared owls (*Asio otus*), and northern saw-whet owls (*A. acadicus*) were collected opportunistically in 1999 during nocturnal, systemic broadcast Mexican spotted owl surveys conducted in 25 study areas or quadrats of varying size scattered across Central Arizona and into New Mexico [48]. Surveys were solely aimed at detecting Mexican spotted owls. However, non-target owls are often heard or seen during nocturnal surveys, with some species even known to respond to heterospecific call broadcast [49,50]. A few of the locations were visual sightings, but most were auditory detections. Surveys were conducted from georeferenced call points. If an owl was very close to a point, observers recorded the call point location as the owl location. For more distant owls, observers estimated the distance and bearing to the calling owl from the call point and then recorded the spatial coordinates from an estimated map location based on that distance and bearing. A total of 303 separate detections were recorded, mostly of great horned owls and flammulated owls, but also including smaller numbers of some of the other owls included in this study. The locations all represented calling owls, not nests. The sampling effort was similar in that call points were generated in each quadrat to ensure complete survey coverage, and multiple surveys were completed at all call points. The total number of complete surveys varied from 3 to 6 per quadrat, reflecting the degree of difficulty of completing surveys (access was more difficult in roadless quadrats). Although heterospecific call broadcast has variable effects on detectability across owl species [50], it likely would not produce any bias in detectability among habitats within species.

A total of 262 detections were recorded within the 25 quadrats. Within a quadrat, multiple locations may have corresponded to a smaller number of unique individuals or territories, constraining the strength of inference regarding habitat associations. In one quadrat with multiple northern saw-whet owl detections, we created 250 m buffers around each detection point in ArcMAP to limit overlap with adjacent points and generate what we then considered separate samples of habitat use. In the Sierra Nevada, Groce and Morrison [51] used the same approach to obtain a general sense of habitat associations at a scale greater than a nest or roost site.

#### 2.3.4. Great Horned Owl Detections (New Mexico)

Great horned owls are known to readily respond to heterospecific call broadcast [49]. During spotted owl surveys in South-Central New Mexico’s Sacramento Mountains from 2003 to 2006 [52], great horned owl detections were opportunistically also recorded (J. Ganey, unpubl. data). The study area covered approximately 50,000 ha at elevations ranging from 2000 to 2800 m. The terrain consisted primarily of heavily forested montane slopes and minor drainages, with interspersed meadows in the larger valley bottoms. The predominant forest type was mixed conifer, singularly or co-dominated by white fir (*Abies concolor*) and Douglas fir, with more limited extents of ponderosa pine forest. All recorded locations (*n* = 45) represented calling owls, and most detections were auditory, with a few visual observations.

#### 2.3.5. Whiskered and Western Screech-Owl Nest Box Study (Arizona)

A nest box study was conducted during the breeding seasons of 2015–2022 in Miller Creek Canyon in the Peloncillo Mountains in Southeastern Arizona [53]. The elevation ranged from 1553 to 1610 m. The vegetation within the study area was dominated by an oak overstory and a mixed understory of alligator juniper (*Juniperus deppeana*), pointleaf manzanita (*Arctostaphylos pungens*), and catclaw acacia (*Senegalia greggii*). Through mist netting captures, both whiskered screech-owls (*M. trichopsis*) and western screech-owls were shown to be present in the study area before nest boxes were deployed. A total of 15 installed nest boxes were monitored for occupancy and nesting success [53].

### 2.4. Spatial Model Validation

Validation is the most challenging task in the habitat modeling process, with no single test or approach accepted as the gold standard in assessing model performance [54,55]. Compositional procedures have been recommended for the validation of habitat models in the absence of ad hoc presence and absence datasets [55]. We used Arc GIS Pro and a spatial randomized sampling procedure to test the performance of our species habitat profiles and habitat maps under current conditions. Within an area corresponding to all woodland and forest types (woodland, montane, and subalpine zones; Table 1), we randomly generated sample points equal in number to the occurrence records for a given species in our validation dataset. To exclude areas where species were known to be absent, the sampling procedure was constrained geographically for the whiskered screech-owl (Sky Island–Bootheel ecoregion; Figure 2), boreal owl (San Juan–Sangre de Cristo and Jemez Mountains ecoregions), and northern saw-whet owl (all ecoregions except for the Sonoran ecoregion). Using the Python function *arcpy.analysis.Intersect*(), we then calculated the number of random points intersecting with polygons representing the predicted habitat of the species (Supplementary Materials). For every species, the randomization procedure was repeated for a total of 500 iterations before applying one-tailed z-tests with continuity corrections to test the null hypothesis, “the proportion of occurrence records captured by the predicted habitat model is not significantly greater than that of a randomly generated sample” (Supplementary Materials). Z-tests were conducted in R 4.3.1 (R Core Team 2022) using *Tidyverse* (R programming package) and the proportions of records falling within the predicted mapped habitat with and without the 1 km buffer (designed to account for spatial error in mapping and occurrence records such as species recorded in grassland within 1 km of a woodland or forest polygon).

### 2.5. Assessment of Climate Change Impacts

For each of the eight species, we conducted McNemar tests in R 4.3.1 for paired, binary response data to test the null hypothesis that the proportion of records or detection points within vs. outside the 2017 mapped modeled habitat does not differ between current and projected climate-induced conditions. McNemar tests are preferred over Chi-squared tests to determine whether there is a significant change in nominal data before and after an event (in this case, climate change), based on repeated observations of the same individuals, with data presented in a 2 × 2 table showing the numbers of concordant vs. discordant cells, instead of the Chi-squared test’s contingency table [56]. Tests were performed using the R base function *mcnemar.test*() and repeated with and without the 1 km buffer around the mapped habitat (Supplementary Materials). McNemar’s continuity correction was applied whenever any of the cell counts in the 2 × 2 matrix were less than 5.

## 3. Results

### 3.1. Spatial Model Validation

In terms of omission errors, our current (2017) habitat model performed well for five species, with 100% of georeferenced records captured within the buffer for the boreal owl and whiskered screech-owl, 98.6% for the northern pygmy-owl, 95.6% for the flammulated owl, and 93.2% for the great-horned owl (Table 2). Performance was lower for the northern saw-whet owl (82.4% captures within the buffer), long-eared owl (78.3%), and western screech-owl (53.3%). For all eight species, the use of the 1 km buffer improved the performance of our habitat model. Some records fell just outside the mapped habitat, as was the case for two of the three boreal owl records not directly captured by the model but within less than 100 m of the mapped extents (and thus captured inside the buffer). Without applying the buffer, as many as 88 (34%) of all 256 records outside the mapped habitat fell in riparian areas nestled within woodlands and forests, with another 48 (19%) in montane/subalpine and other grasslands. For the whiskered screech-owls, 23 riparian records represented the majority (56%) of all whiskered screech-owl records falling outside the mapped habitat. Of the total number of northern pygmy-owl records outside the mapped habitat, 16 (73%) also occurred in riparian areas. Smaller numbers of records mapped in upland shrub, including eight flammulated owl records falling in the Mojave–Sonoran Desert Scrub, evidently represented mapping errors, which were limitations of the validation dataset.

Without applying the 1 km buffer, the habitat models significantly outperformed randomized samples (generated from woodlands and forests) in the case of the flammulated owl, northern pygmy-owl, long-eared owl, boreal owl, and northern saw-whet owl; results for the western screech-owl were marginally significant (Table 2). With the 1 km buffer included, the western screech-owl and whiskered screech-owl habitat models also significantly outperformed the random sampling procedures. Overall, it becomes increasingly more difficult for a habitat model to outperform randomization procedures as the number of woodland forest ERUs in the habitat profile increases. Because all woodland and forest types were included in the great horned owl habitat profile, all random points (from woodland and forest types) necessarily intersected with that profile. The lack of significant results for the great horned owl thus does not mean that its habitat model is flawed. Nonetheless, greater caution is needed in interpreting model projections for this species, in addition to the western and whiskered screech-owls, given the likelihood of greater uncertainty in the results.

### 3.2. Climate Change Projections

**Figure 3:**
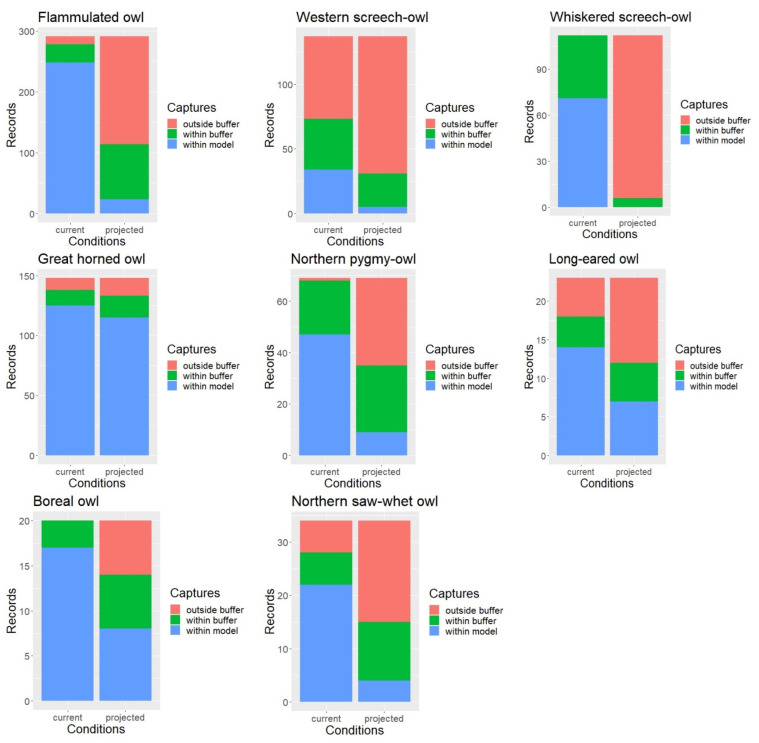
Model validation results and difference in the proportion of records within vs. outside mapped habitat under current and projected conditions.

Under the current conditions, approximately 88% of all owl survey records were captured by the habitat profile mapping when allowing for a 1 km buffer to account for spatial accuracy in the data. When intersected with the late 21st century ERU projections, only about 43% of the owl records or detection points fell within the modeled habitat (with the 1 km buffer), with percent capture values ranging between 5% for the whiskered screech-owl and nearly 90% for the great horned owl but overall much lower than under the current conditions for nearly every species (Figure 3).

When using the 1 km buffer, the McNemar’s test results showed that the proportion of owl record locations falling outside our spatial model was indeed significantly higher under the projected future conditions compared to the current conditions for all but one species (Table 3). Results for the great horned owl were only marginally significant. Without applying the 1 km buffer, the proportion of owl records falling outside our mapped habitat was significantly higher under the projected conditions for all eight owl species.

**Figure 4:**
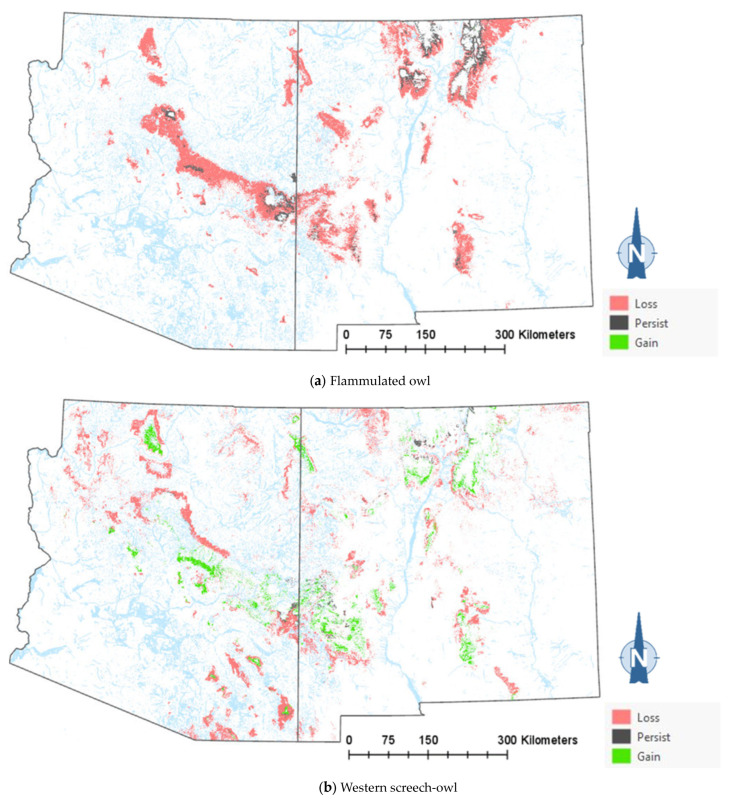

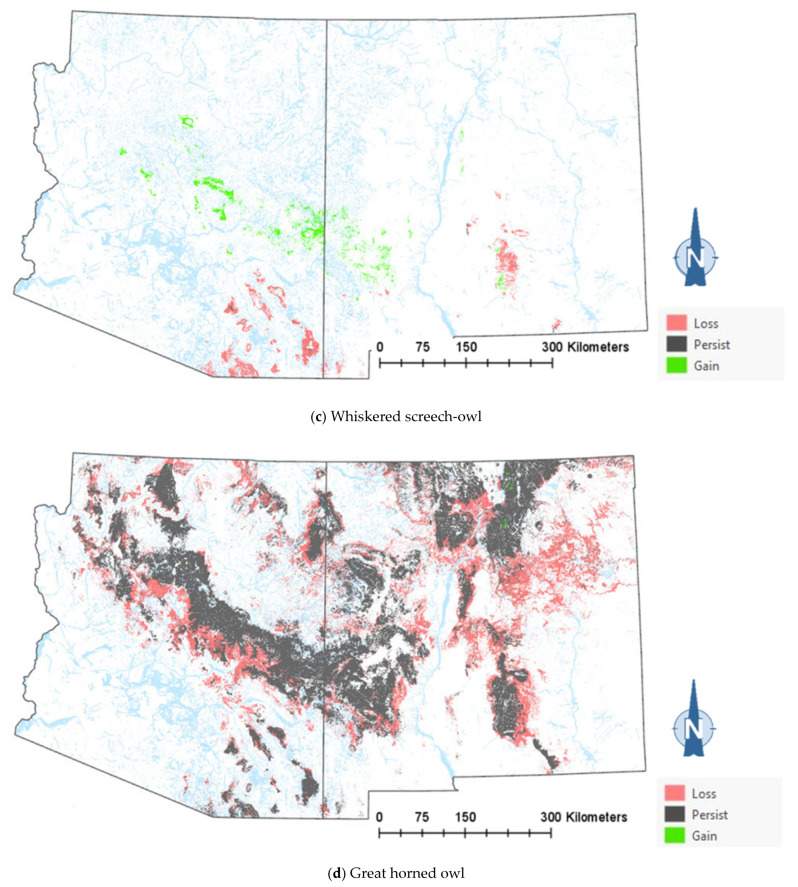

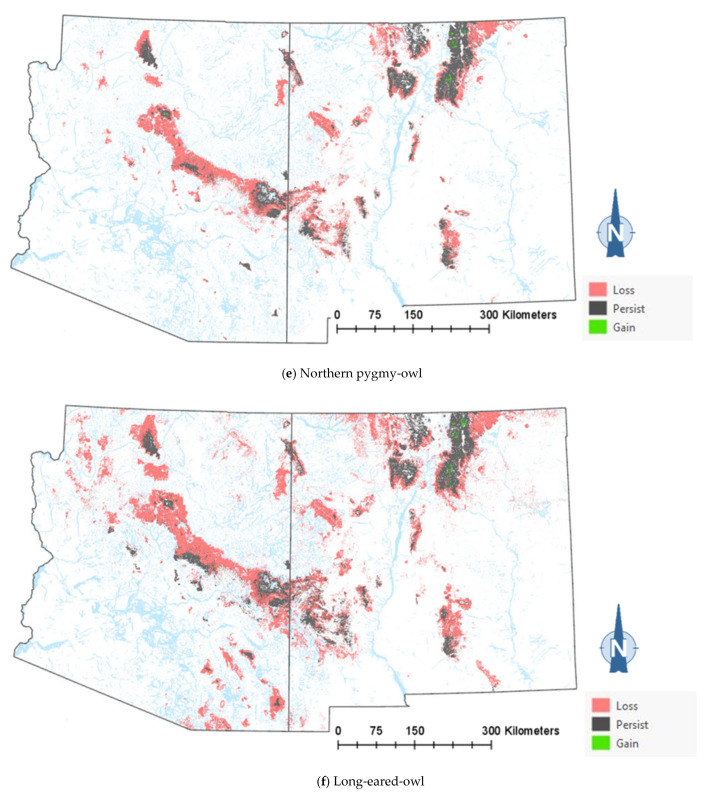

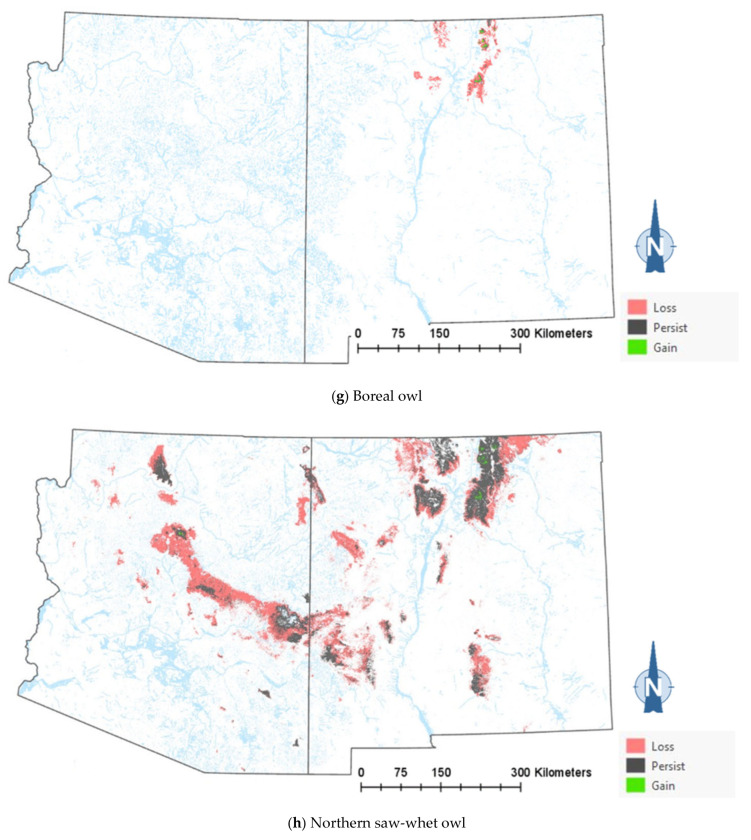
Distribution of modeled habitat for eight owl species under current vs. 2090 projected conditions, Southwestern US. Modeled habitat under 2090 projected conditions is the sum of the modeled current habitat that has persisted or been gained.

The comparison of the current and projected distribution of modeled owl habitats indicated range contractions within the region for all eight owl species, with mid- to upper-elevation habitat declines varying from −5% to −86%, and with the boreal and flammulated owls predicted to have the greatest habitat loss (Figure 4, Table 4). The habitat range of one species, the whiskered screech-owl, was projected to expand northward, nearly offsetting the losses in habitat extent over the long term, with an estimated decrease of only 5%. Wherever habitat gains were projected (mostly observed for the whiskered and western screech-owls), the new mapped habitat was typically not adjacent to the existing mapped habitat. The current mapped habitat for the boreal owl (all in Northern New Mexico) consisted of 899 dissolved polygons (i.e., separate patches) ranging in size from 0.12 to 80,179 ha ($\bar{x}=$ 331.39 ha ± 3146.82). The projected 2090 mapped habitat was reduced to 311 separate patches averaging 133.59 ha ± 615.22, with a maximum size of just under 6128 ha. The eight boreal owl records still within the mapped habitat under the projected future conditions were located within five patches totaling 6401 ha ($\bar{x}=$ 1280 ha, range = 11.3–3563 ha), compared to six patches totaling 149,285 ha ($\bar{x}=$ 24,881 ha, range = 4162–80,179 ha) under the current conditions.

### 3.3. Flammulated Owl Breeding Populations

**Figure 5:**
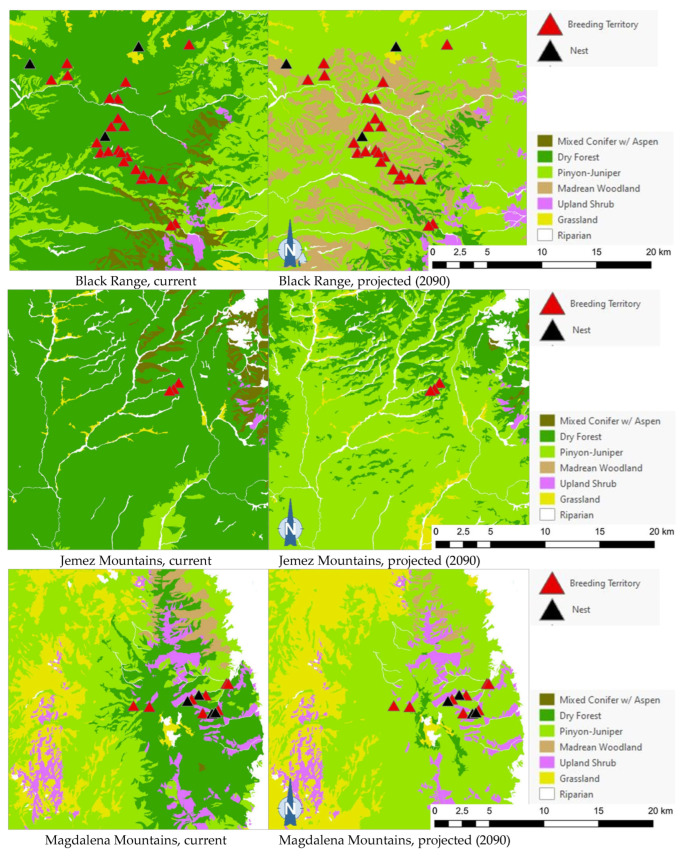

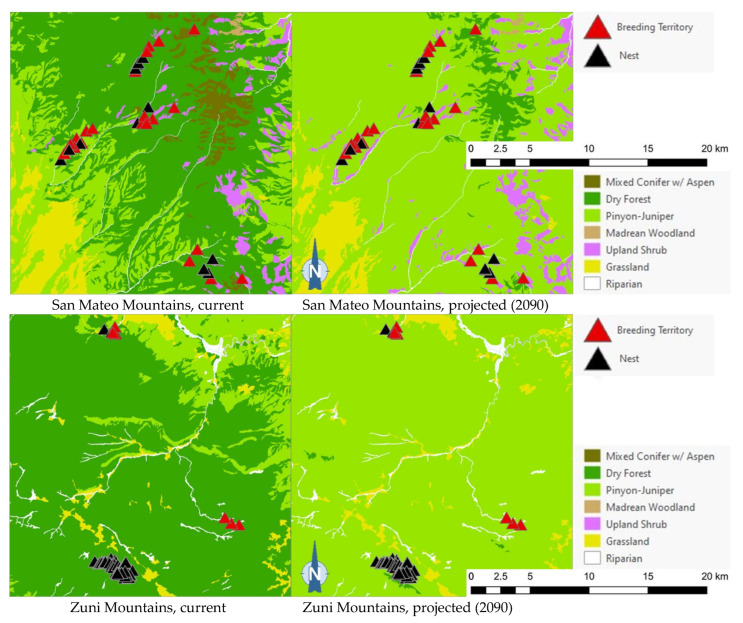
Distribution of flammulated owl territories (red triangles) and nests (black triangles) across current and 2090 projected general vegetation types in the Black Range and in the Jemez, Magdalena, San Mateo, and Zuni Mountains of New Mexico. In the Zuni Mountains, all 72 recorded flammulated owl territories and nests were mapped in dry forest (ponderosa pine, pine-oak, and frequent-fire mixed-conifer forest) under current conditions; vegetation shifts projected by 2090 placed all 72 territories and nests in pinyon–juniper instead, outside the species’ habitat profile.

The projected flammulated owl habitat shifts affecting 148 documented breeding territories across five mountain ranges of New Mexico ([32], D. Arsenault, unpubl. data) are summarized in Table 5 and shown in Figure 5. About 92% of territories were found to currently fall within the expected habitat profile of flammulated owls, with a strong preference for dry forests (ponderosa pine, pine oak, and frequent-fire mixed conifer forests). Three territories occurred in pinyon–juniper stands within 600 m or less of dry forest and leaving open the possibility of spatial uncertainty in vegetation type mapping or territory records. Nine documented territories occurred in nearby riparian corridors or shrub types, all within 200 m of dry forest. Late 21st century habitat projections suggested that the climate conditions in over 80% of territories currently suitable for owl habitats will favor the development of pinyon–juniper and Madrean woodland types, with the possibility of some territories falling within upland shrublands and less than 10% of the current owl territories remaining within the present concept of flammulated owl habitats.

Of the five flammulated owl populations analyzed, four were projected to face vegetation shifts placing all or nearly all current nesting territories outside the species’ habitat profile, whether pinyon–juniper woodland, Madrean woodland, or upland shrub (Figure 5). The only exception was the Jemez Mountains flammulated owl population, which would still find suitable vegetation where nesting territories currently exist.

### 3.4. Northern Saw-Whet Detection Points

**Figure 6:**
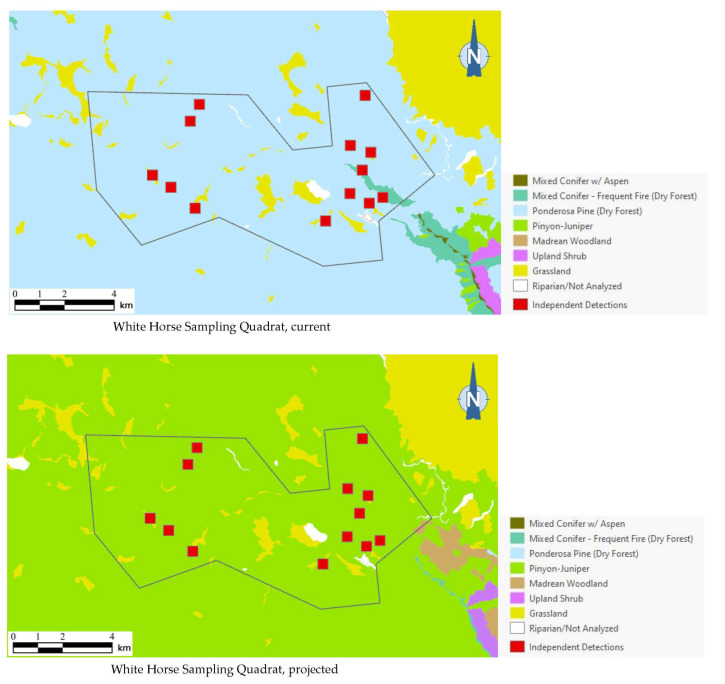
Distribution of northern saw-whet owl detection points (red squares) among current and 2090 projected general vegetation types in the White Horse Sampling Quadrat on Arizona’s Coconino Plateau. All 13 northern saw-whet owl 1999 detection points were mapped in ponderosa pine forest under current conditions; vegetation shifts projected by 2090 placed these same detection points in pinyon–juniper instead, outside the species’ habitat profile.

As the best approximation of habitat use by northern saw-whet owls in any given area (based on our dataset), or even of the existence of a breeding population, a total of 13 independent detection points (i.e., detection points with non-overlapping, 250-m-diameter buffers around them) for this species occurred in one sampling quadrat in Arizona. Of the 13 independent detection points, 12 (92%) were mapped within ponderosa pine forest, which covered 93% of the quadrat’s area (Figure 6). Under the 2090 projections, however, all of these detection points were mapped outside the species’ habitat profile.

## 4. Discussion

Differences between the current and future habitat distribution reflect a 21st-century departure from historical climate envelopes for the significant underlying features of vegetation types at any given location. Modeled habitat distributions suggest substantial changes in familiar ecosystem conditions and, in particular, significant losses in montane woodland and forest habitats for most owl species evaluated with this study. This forecast does not consider disturbance processes such as wildfires, broadly considered catalysts for the rapid and permanent type conversion of vegetation under this century’s climate trends [57].

The modeling results show that climate-driven range contractions can be expected in the Southwestern US for several owl species. Of those species assessed, the boreal owl is arguably the most sensitive to warming trends as trailing-edge populations (near the low-latitude edge of the distribution) are generally predicted to face the highest risk of climate-induced extinction [58]. The southernmost part of the boreal owl’s range narrowly extends into three mountain ranges in Northern New Mexico [46,47], and the 2090 projected conditions include the complete loss of historical habitats in one of these mountain ranges, in addition to severe habitat losses in the other two ranges (Figure 4). Particularly concerning are our projections of important reductions in the size of future, individual mapped habitat areas for the boreal owl in New Mexico. Summer home range areas in Idaho were found to average 1182 ± 335 ha for 15 owls, with an observed minimum of 229 ha and an observed maximum of 2386 ha [59]. Although, in Idaho, boreal owls were not restricted to spruce–fir forest (they nested in mixed-conifer forest, where they presumably found more suitable nesting cavities, but roosted and foraged in spruce–fir forest), they also selected cool roosting sites during hot weather periods, when they often exhibited signs of heat stress [59]. Our projections of only four future habitat area patches exceeding the maximum home range size documented in Idaho (and only 8 and 26 greater in size than the observed average and minimum home range, respectively) raise the possibility that under future conditions in New Mexico, the boreal owl will become extirpated from the state. To persist in New Mexico, any remaining boreal owls would likely need to maintain smaller home ranges or increase their relative use of lower-elevation vegetation despite an increased likelihood of heat stress. Under one possible scenario, Northern New Mexico would function as a sink habitat. Some patches of spruce–fir forest would continue to be occupied, but only with an influx of immigrants from Colorado’s high mountainous terrain to the north. Another possibility is that those few extents that currently exist as alpine would gradually transition to spruce–fir forest from its leading edge, where it advances upwards with increasing temperatures and as climate conditions become less favorable for alpine. Here, there is an expected lag in the advance of spruce–fir forest relative to the rate of decline at the lower trailing edge, consistent with observations elsewhere in the region [38].

Warming trends and increased aridity are not only likely to reduce the amount of owl habitats in the region but also favor population shifts northward and upward in elevation within the region. Indeed, model outputs for the boreal, western screech, and whiskered screech-owls (Figure 4a–h) corroborate a theorized northward shift in habitat conditions for some species. Outputs for boreal, flammulated, long-eared, and northern pygmy-owls support expectations that habitats will shift upward in elevation. Any shift north in habitat for the whiskered screech-owl may represent a range expansion, though the species will nonetheless likely experience a net loss of habitat with ongoing climate trends (Table 4).

### 4.1. Model Uncertainty

There is always uncertainty in model outputs, as with the results reported here on habitat distribution and decline. The model validation showed that the habitat profile tested was spatially inclusive of all survey data when a buffer was applied, and that model parameterization may reflect a reasonable balance of omission and commission error. Nevertheless, greater confidence may be warranted for results for the percent change in habitat conditions (current to future) versus specific area estimates in Table 4. Uncertainty in the underlying climate vulnerability projections tends to be greater in mountainous terrain and lower in the basins and plains of the region [17], with the implication that habitat models associated with upper life zones may have added uncertainty—i.e., for the boreal, northern saw-whet, and flammulated owls. Although not included in our modeling, nest locations for the long-eared, western screech, and great horned owls can also occur in low-elevation riparian areas separate from suitable upland forest and woodland habitats. The ability to include riparian habitats in future habitat models could improve the predictive ability and usefulness of model outputs. Riparian and canyon habitats represented in some survey records for the western screech-owl, habitat features not expressed in its model profile, help to explain the lower validation results for this species. These valley bottom settings usually possess upland vegetation, including that of cooler and moister life zones of higher elevation, given how riparian zones and canyon bottoms are protected from sun exposure and how they concentrate moisture. In addition to the great horned owl, the western screech-owl is known to breed in low-elevation areas such as the Sonoran Desert in Arizona [29,30]. Ongoing trends and 21st-century forecasts for temperature and aridity in the region [60,61] suggest that desert regions may increase in extent [62].

### 4.2. Vegetation vs. Resource Habitat Models

Resource-based habitat models have been shown to perform better than vegetation-based habitat models for some species [63,64]. Although our spatial model functioned well for most of the owl species included in this study, they likely did not fully capture the spatial occurrence, or lack thereof, when scaled over space and time. The northern saw-whet owl tends to occur sporadically throughout its distribution, being locally common in some years and absent in others, perhaps tracking irruptive populations of small mammals, an important, temporal habitat component [65]. High prey densities are also believed to be key for the long-eared owl, often referred to as a nomadic breeder [66]. Nest cavities may represent another important limiting resource in the flammulated owl, western and whiskered screech-owls, northern pygmy-owl, boreal owl, and northern saw-whet owl, all secondary cavity nesters. In New Mexico, for example, areas with suitable nest cavities were found to be saturated with flammulated owl territories [32]. Cavities large enough for flammulated owls in New Mexico are those excavated by the acorn woodpecker (*Melanerpes formicivorus*) and the northern flicker (*Colaptes auratus*), but the distribution of the latter species is largely limited by the availability of soft wood in the form of ponderosa pine snags and large quaking aspens [32,67].

### 4.3. Fire

There is increasing evidence of a shrinking forest–woodland extent in the west and shifting vegetation types [34,37] with upward changes in elevation at an average rate of 15 m or more per decade [35,38,39]. Concerns about shifts in vegetation types and related habitat conditions are heightened by the potential for abrupt and lasting change with severe wildfires [68]. Of particular concern are the combined effects of warming, drought, and wildfires on forests and woodlands [69,70] and the compounding effects of fire exclusion and unnaturally high tree densities in fire-adapted forests and woodlands [71]. From 2011 to 2013, two wildfires in the Jemez Mountains (one of the three mountain ranges harboring the totality of New Mexico’s boreal owl population) totaling > 700 km^2^ reduced the extent of spruce–fir forest around the southern and eastern portions of the Valles Caldera National Preserve by 34 km^2^ [47]. During these same three years, three fires in the southern Sangre de Cristo Mountains burned through 54 km^2^ of spruce–fir forest. In 2013, one of these fires burned through one of the boreal owl’s historical locations [47]. The 2022 Calf Canyon/Hermits Peak Fire became the largest fire in the state of New Mexico’s history after burning through 135,934 hectares in the Sangre de Cristo Mountains, of which 29,604 hectares (22%) corresponded to spruce–fir forest. As no boreal owl surveys were ever conducted in the spruce–fir forest stands affected by the 2022 Calf Canyon/Hermits Peak Fire (D. W. Stahlecker, unpubl. data), we cannot rule out the loss of more boreal owl historical habitats, estimated at a maximum of only 306,000 ha in New Mexico (Table 4). Another species that may stand to lose from the increased incidence of severe fires and lack of forest regeneration is the northern saw-whet owl, which showed the near complete avoidance of burned areas in the Pacific Northwest [72].

### 4.4. Adaptation

Some mechanisms to cope with climate change have already been documented in owls and other bird species. Research conducted in Finland used 28 years of past climate data to also link milder winter conditions with the disappearance of selective predation pressure against the tawny owl’s brown morph (determined largely through genetic inheritance and considered generally more cryptic when there is less snow), an example of adaptive capacity through microevolution [73]. The adaptive capacity of bird populations to warming trends is also revealed by uphill movement to cooler, moister, and more familiar climate conditions, with short-lived species holding an advantage in relocating more quickly [74]. If this evidence applies to the owls of the southwest, the northern saw-whet, flammulated, and northern pygmy-owls may possess some advantage in coping with rapid warming trends relative to longer-lived species such as the long-eared owl. Throughout much of New Mexico, however, flammulated owl populations breeding in ponderosa pine and/or mixed conifer forest may already find themselves on the highest slopes within the same mountain range (e.g., Zuni Mountains). At the same time, flammulated owls likely have good potential for adaptability because they are long-distance migrants with low natal philopatry, making them able to colonize isolated habitats [75].

Though a long-lived species and projected by us to lose 55% of its mapped upper elevation habitat (due to conversion to non-woodland types), the great horned owl may nonetheless be uniquely suited to adapt to projected future climate change. It is known to occupy, and breed in, a very broad range of vegetation types, from desert to subalpine or boreal forest [76]. It exhibits much size and other phenotypic variation [77], can switch between being a prey specialist and a generalist [78], and is capable of long-distance movements following habitat quality reductions [79]. Though much remains to be learned about natal dispersal in the western and whiskered screech-owls, these two species may be characterized by lower adaptive capacity as adults typically remain within the same breeding territories year-round in the Southwestern US. Our study shows that gains in habitat will likely not be enough to offset habitat losses, not to mention that these gains will also not happen in areas neighboring existing habitats. Western and whiskered screech-owls may still be able to shift their distribution through natal dispersal, which remains poorly known. In Southwestern Idaho, juvenile western screech-owls were found to disperse an average of 10.6 ± 1.8 km from natal to overwintering sites, with possible further dispersal later to first breeding sites [80].

### 4.5. Mitigation

The directional change in elevation and latitude that is anticipated for wildlife species and their habitats can help to set priorities for land management aimed at climate adaptation. The repeat inventory of historical vegetation samples in the southwest corroborates expectations for rapid warming trends and habitat responses [35,38,39]. Wildlife specialists and natural resource managers can evaluate the likely shifts among vegetation types under increased warming and aridity, assess the sensitivity and adaptive capacity of plant dominants that make up the habitat structure, and evaluate ongoing owl population trends. The legacy mapping of vegetation and wildlife habitats should not be viewed as static and will challenge practitioners and applied scientists to adapt conventional roles for the interpretation, communication, and guidance to others on the use of spatial information.

### 4.6. Broader-Scale Implications

Most of the world’s owl species now appear to be experiencing global population declines [81]. High exposure and high sensitivity to ongoing and future climate change have been shown in high-latitude owls in particular [8,10,11,82]. Our research points to what may represent an important loss of habitat not just in the Southwestern US but perhaps also in many mountainous regions of arid Southwestern North America and the arid zones of the Subtropics. With climate having a powerful influence on the distribution of biota around the world [83,84], tropical mountain regions have long been known as biodiversity hotspots [85], but there is also increasing recognition of the ecological distinctiveness of subtropical, arid-zone mountain ranges [86,87,88]. Unlike at more temperate latitudes, arid-zone mountains tend to harbor biota distinct from neighboring lowlands, reflecting their role as refugia during past periods of warming temperatures and drying conditions [86,87]. These refugia are now threatened by anthropogenic climate change, with the taxa that they harbor facing range contractions [88] and increased isolation [89]. Added to the projected loss of refugia habitats in arid-zone subtropical regions are findings linking likely future population declines and more extreme temperatures and/or reduced precipitation, not just for the Mexican spotted owl in Southwestern North America [9] but also for the tawny owl in Israel [90].

## 5. Conclusions

We project severe, region-wide breeding habitat losses for most montane owls in the Southwestern US by 2090. The near complete loss of current breeding habitats is projected in particular for known flammulated owl populations in Western New Mexico, while the projected boreal owl habitat distribution will only consist of isolated areas perhaps too small to sustain the regional persistence of the species. Three more species, the northern pygmy-owl, long-eared owl, and northern saw-whet owl, are projected to lose at least 60% of their current breeding habitats. The whiskered screech-owl is predicted to lose all its current habitat and will only persist in the region if it can track areas that become suitable to the north of its current range. More uncertainty exists for two species with distributions that extend down to lower elevations.

## Figures and Tables

**Table 1:** Ecological Response Units present in foothills and mountains of the Southwestern US (New Mexico and Arizona) and used for species habitat models ^1^.

Vegetation Zone	Ecological Response Unit
Subalpine Zone	Spruce–fir forest
	Bristlecone pine forest
Montane Zone	Mixed conifer with aspen
	Mixed conifer—frequent fires
	Ponderosa pine forest
	Ponderosa pine–evergreen oak forest
Woodland Zone	Pinyon–juniper woodland
	Madrean pinyon–oak woodland
	Madrean encinal woodland
	Pinyon–juniper sagebrush
	Pinyon–juniper evergreen shrub
	Pinyon–juniper deciduous shrub
	Pinyon–juniper grass
	Juniper grass

^1^ Though they represent additional Ecological Response Units, riparian systems were excluded from the species habitat models as they could not be modeled under projected future (2090) conditions.

**Table 2:** Habitat model performance and results of spatial randomized sampling procedures and 1-tailed z-tests to accept or reject the null hypothesis that the predicted habitat captured a proportion of occurrence records that was not significantly greater than that of a randomly generated sample from within woodlands and forests ^1,2^.

Species	No. Records				No. Simulations Equaling or Outperforming Habitat Model	
	Within Model	Within Buffer	Outside Buffer	Total	Without the 1 km Buffer	With the 1 km Buffer
Flammulated owl	248	30	13	291	0 ****	0 ****
Western screech-owl	34	39	60	137	34 ^(^*^)^	0 ****
Whiskered screech-owl	71	41	0	112	307	0 ****
Great horned owl	125	13	10	148	500	500
Northern pygmy-owl	47	21	1	69	0 ****	0 ****
Long-eared owl	14	4	5	23	17 *	0 ****
Boreal owl	17	3	0	20	0 ****	0 ****
Northern saw-whet owl	22	6	6	34	0 ****	0 ****

^1^ Randomized sampling results were based on 500 iterations for every species. ^2^ Significant *p*-values are indicated in bold; **** *p* < 0.0001, * *p* < 0.05, ^(^*^)^ *p* < 0.1.

**Table 3:** Results of McNemar tests (Chi-square values) to accept or reject the null hypothesis that the proportion of records or detection points within vs. outside the 2017 mapped modeled habitat did not differ between current and projected climate-induced conditions ^1^.

Species	No. Observations	Mapped Habitat			
		With the 1 km Buffer		Without the 1 km Buffer	
		χ^2^	*p*-Value	χ^2^	*p*-Value
Flammulated owl	291	**162.01**	<0.0001 ****	**223**	<0.0001 ****
Western screech-owl	137	**40.02**	<0.0001 ****	**27.03**	<0.0001 ****
Whiskered screech-owl	112	**104.01**	<0.0001 ****	**69.01**	<0.0001 ****
Great horned owl	148	3.2	0.07 ^(^*^)^	**8.1**	0.004 **
Northern pygmy-owl	69	**31.03**	<0.0001 ****	**36.03**	<0.0001 ****
Long-eared owl	23	**4.17**	0.04 *	**5.14**	0.02 *
Boreal owl	20	**4.17**	0.04 *	**7.11**	0.008 **
Northern saw-whet owl	34	**11.08**	0.0008 ***	**16.06**	<0.0001 ****

^1^ Significant *p*-values are indicated in bold; **** *p* < 0.0001, *** *p* < 0.001, ** *p* < 0.01, * *p* < 0.05, ^(^*^)^ *p* < 0.1.

**Table 4:** Current and projected habitat area for each owl species analyzed, along with percent change in extent.

Species	Vegetation Types Representing Preferred or Primary Habitat	Owl Habitat Model Zones (Figure 1)	Approx. Current 2017 Extent (ha)	Approx. Projected 2090 Extent (ha)	Projected 2090% Change
Flammulated owl	Montane forests	Regionwide	4,684,000	714,000	−85%
Western screech-owl	Pinyon–juniper and Madrean woodlands	Regionwide	3,186,000	1,511,000	−55%
Whiskered screech-owl	Ponderosa and ponderosa pine–evergreen oak forest, and Madrean woodlands	Sky Island—Bootheel, Pecos East (Guadalupe Mtns only), AZ-NM Mtns (2090 only), Pecos West (2090 only)	714,000	677,000	−5%
Great horned owl	All forest and woodland types	Regionwide	16,984,000	11,273,000	−35%
Northern pygmy-owl	Subalpine and montane forests, pinyon–juniper and Madrean woodlands	Regionwide	8,267,000	2,021,000	−75%
Long-eared owl	Subalpine and montane forests, pinyon–juniper and Madrean woodlands	Regionwide	8,263,000	2,623,000	−70%
Boreal owl	Subalpine forests	Sangre de Cristo, San Juan, and Jemez Mountains	306,000	42,000	−85%
Northern saw-whet owl	Subalpine and montane forests	Regionwide except Sonoran zone	5,080,000	2,008,000	−60%

**Table 5:** Number of flammulated owl sample territories among current and projected vegetation types in the Black Range and the Jemez, Magdalena, San Mateo, and Zuni Mountains in Western New Mexico.

	Projected Vegetation Type (to)					
Current Vegetation Type (From)	Dry Forest	Madrean Woodland	Pinyon–Juniper	Riparian	Gambel Oak Shrubland	Total
Dry Forest ^1^	1	9	122			132
Mixed Conifer with Aspen	4					4
Pinyon–Juniper			2		1	3
Riparian				7		7
Upland Shrub					2	2
Total	5	9	124	7	3	148

^1^ Dry forest consists of Ponderosa Pine Forest, Ponderosa Pine–Evergreen Oak Forest, and Mixed Conifer/Frequent Fire combined.

## Data Availability

Georeferenced, museum specimen records from New Mexico and Arizona can be publicly queried at https://arctos.database.museum/search.cfm (accessed on 29 November 2023); i-Naturalist records are publicly available at https://www.inaturalist.org/observations (accessed on 29 November 2023). The validation dataset is available from the authors upon request.

## Supplementary Materials

The Python and R code used for this study, as well as the results of the McNemar and z-tests, can all be downloaded at https://github.com/mcartron10/Owl-Research.git (accessed on 4 December 2023).
